# Supplementary material for: Transient nicotine exposure in early adolescent male mice freezes their dopamine circuits in an immature state
Source: Nat Commun. 2024 Oct 18;15:9017. doi: 10.1038/s41467-024-53327-w (PMC11489768; doi:10.1038/s41467-024-53327-w)
Supplement: Supplementary file 1 — Supplementary Information [file 41467_2024_53327_MOESM1_ESM.pdf]

Supplementary information

Transient nicotine exposure in early adolescent male mice freezes their dopamine circuits in an immature state

Supplementary Figure 1. *Supplementary data for Figure 1*. (**A**) Individual weight measurements across the entire oral self-administration task for adolescent-pretreated (*Left*) and adult-pretreated mice (*Right*). (**B**) Actograms of drinking behavior over the duration of the Two Bottle Choice task for adult mice pre-treated in adolescence (*left*) or in adulthood (*right*). Shaded regions represent mean volumes across the groups for each solution tested. (**C**) Sucrose solution and overall liquid consumption per day for adolescent-pretreated (*Left*) and adult-pretreated mice (*Right*) during the Sucrose nicotine oral self-administration testing. (**D**) Nicotine solution and overall liquid consumption per day for adolescent-pretreated (*Left*) and adult-pretreated mice (*Right*) during the nicotine oral self-administration testing. (**E**) Individual data for Figure 1B *center*, % nicotine of total intake for mice treated with NIC or SAC in adolescence. (**F**) Individual data for Figure 1C *center*, % nicotine of total intake for mice treated with NIC or SAC in adulthood. (**G**) Percent preference for left side bottle across the entire oral self-administration task for adolescent-pretreated (*Left*) and adult-pretreated mice (*Right*). (**H**) Individual data for Figure 1E, solid lines indicate group means while dotted lines are individual animals for mice treated with NIC or SAC in adolescence. (**I**) Open arm entries (*Left*) and Distance traveled (cm, *Right*) in the EOM task for adolescent-pretreated mice. (**J**) Individual data for Figure 1H, solid lines indicate group means while dotted lines are individual animals for mice treated with NIC or SAC in adulthood. (**K**) Open arm entries (*Left*) and Distance traveled (cm, *Right*) in the EOM task for adult-pretreated mice. All line graphs are presented as mean values ± SEM. *p < 0.05, **p < 0.01, ***p < 0.01, ns = not significant. Detailed Statistics are available in Supplementary Table 4. Source data are provided as a Source Data file.

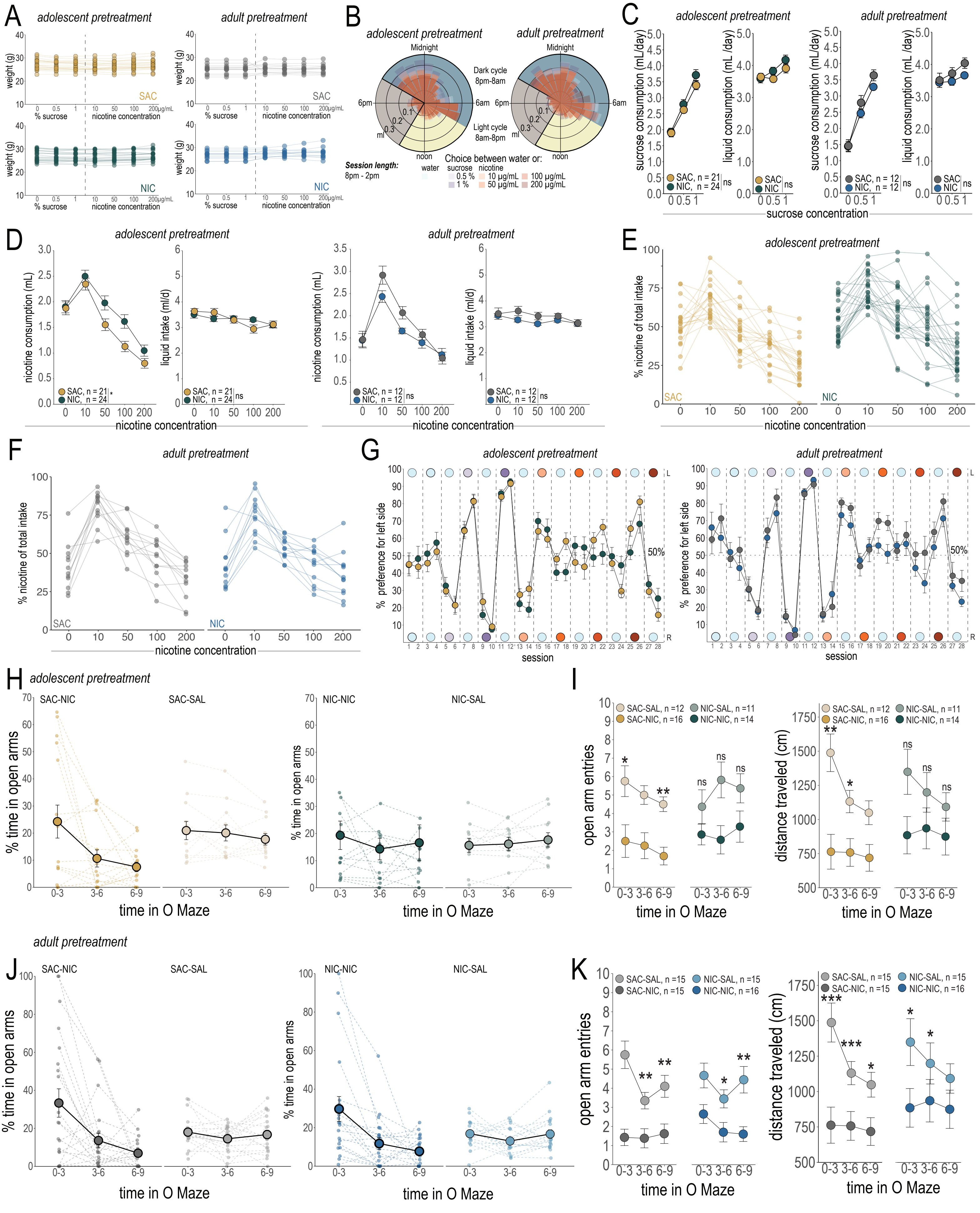

Supplementary Figure 1

Supplementary Table 1

Detailed Statistics for Supplementary Figure 1

N	Test	Factor	Statistic	Statistic Value	p value	Corresponding Figure
**weight - adolescents**						
SAC = 21, NIC = 24	Shapiro-Wilk normality test		W	0.9723	0.0000	Fig S1A
	Wilcoxon rank sum test with continuity correction	SAC vs NIC at 0%	W	237.0000	1.0000*	
	Wilcoxon rank sum test with continuity correction	SAC vs NIC at 0.5%	W	250.5000	1.0000*	
	Wilcoxon rank sum test with continuity correction	SAC vs NIC at 1%	W	256.5000	1.0000*	
	Wilcoxon rank sum test with continuity correction	SAC vs NIC at 10µg/mL	W	264.5000	1.0000*	
	Wilcoxon rank sum test with continuity correction	SAC vs NIC at 50µg/mL	W	256.5000	1.0000*	
	Wilcoxon rank sum test with continuity correction	SAC vs NIC at 100µg/mL	W	249.5000	1.0000*	
	Wilcoxon rank sum test with continuity correction	SAC vs NIC at 200µg/mL	W	251.5000	1.0000*	
**weight - adults**						
SAC = 12, NIC = 12	Shapiro-Wilk normality test		W	0.9771	0.0070	Fig S1A
	Wilcoxon rank sum test with continuity correction	SAC vs NIC at 0%	W	60.0000	1.0000*	
	Wilcoxon rank sum test with continuity correction	SAC vs NIC at 0.5%	W	60.0000	1.0000*	
	Wilcoxon rank sum test with continuity correction	SAC vs NIC at 1%	W	60.0000	1.0000*	
	Wilcoxon rank sum test with continuity correction	SAC vs NIC at 10µg/mL	W	71.0000	1.0000*	
	Wilcoxon rank sum test with continuity correction	SAC vs NIC at 50µg/mL	W	75.5000	1.0000*	
	Wilcoxon rank sum test with continuity correction	SAC vs NIC at 100µg/mL	W	81.0000	1.0000*	
	Wilcoxon rank sum test with continuity correction	SAC vs NIC at 200µg/mL	W	75.0000	1.0000*	
**sucrose consumption (mL/day) - adolescent pretreatment**						

* Holm correction for multiple comparisons

Supplementary Table 1

Detailed Statistics for Supplementary Figure 1

N	Test	Factor	Statistic	Statistic Value	p value	Corresponding Figure
SAC = 21, NIC = 24	Shapiro-Wilk normality test		W	0.9223	0.5218	Fig S1C
	Two-way mixed ANOVA	Interaction	F	0.9197	0.4025	
		Treatment (between subj)	F	1.4590	0.2337	
		Sucrose conc (within subjects)	F	119.6943	0.0000	
**total liquid intake (mL/day) - adolescent pretreatment**						
SAC = 21, NIC = 24	Shapiro-Wilk normality test		W	0.8878	0.5218	Fig S1C
	Two-way mixed ANOVA	Interaction	F	1.9069	0.4025	
		Treatment (between subj)	F	1.0621	0.2337	
		Sucrose conc (within subjects)	F	12.6504	0.0000	
**sucrose consumption (mL/day) - adult pretreatment**						
SAC = 12, NIC = 12	Shapiro-Wilk normality test		W	0.9088	0.4287	Fig S1C
	Two-way mixed ANOVA	Interaction	F	0.5978	0.5531	
		Treatment (between subj)	F	2.5198	0.1174	
		Sucrose conc (within subjects)	F	0.0534	0.9480	
**total liquid intake (mL/day) - adult pretreatment**						
SAC = 12, NIC = 12	Shapiro-Wilk normality test		W	0.8711	0.2307	Fig S1C
	Two-way mixed ANOVA	Interaction	F	0.4784	0.6220	
		Treatment (between subj)	F	3.1207	0.0821	
		Sucrose conc (within subjects)	F	1.3045	0.2785	
**nicotine consumption (mL/day) - adolescent pretreatment**						
SAC = 21, NIC = 24	Shapiro-Wilk normality test		W	0.9888	0.0769	Fig S1D
	Two-way mixed ANOVA	Interaction	F	1.7830	0.1344	

* Holm correction for multiple comparisons

Supplementary Table 1

Detailed Statistics for Supplementary Figure 1

N	Test	Factor	Statistic	Statistic Value	p value	Corresponding Figure
		Treatment (between subj)	F	5.8579	0.0198	
		nicotine conc (within subjects)	F	62.9866	0.0000	
**total liquid intake (mL/day) - nic choice - adolescent pretreatment**						
SAC = 21, NIC = 24	Shapiro-Wilk normality test		W	0.9841	0.0127	Fig S1D
	Wilcoxon rank sum test with continuity correction	SAC vs NIC at 0µg/mL	W	243.0000	1.0000*	
	Wilcoxon rank sum test with continuity correction	SAC vs NIC at 10µg/mL	W	205.0000	1.0000*	
	Wilcoxon rank sum test with continuity correction	SAC vs NIC at 50µg/mL	W	274.0000	1.0000*	
	Wilcoxon rank sum test with continuity correction	SAC vs NIC at 100µg/mL	W	312.5000	0.8610*	
	Wilcoxon rank sum test with continuity correction	SAC vs NIC at 100µg/mL	W	259.0000	1.0000*	
**nicotine consumption (mL/day) - adult pretreatment**						
SAC = 12, NIC = 12	Shapiro-Wilk normality test		W	0.9664	0.0043	Fig S1D
	Wilcoxon rank sum test with continuity correction	SAC vs NIC at 0µg/mL	W	243.0000	1.0000*	
	Wilcoxon rank sum test with continuity correction	SAC vs NIC at 10µg/mL	W	205.0000	0.3130*	
	Wilcoxon rank sum test with continuity correction	SAC vs NIC at 50µg/mL	W	274.0000	0.0829*	
	Wilcoxon rank sum test with continuity correction	SAC vs NIC at 100µg/mL	W	312.5000	0.8564*	
	Wilcoxon rank sum test with continuity correction	SAC vs NIC at 100µg/mL	W	259.0000	1.0000*	
**total liquid intake (mL/day) - nic choice - adult pretreatment**						
SAC = 12, NIC = 12	Shapiro-Wilk normality test		W	0.9770	0.0374	Fig S1D
	Wilcoxon rank sum test with continuity correction	SAC vs NIC at 0µg/mL	W	67.0000	1.0000*	

* Holm correction for multiple comparisons

Supplementary Table 1

Detailed Statistics for Supplementary Figure 1

N	Test	Factor	Statistic	Statistic Value	p value	Corresponding Figure
	Wilcoxon rank sum test with continuity correction	SAC vs NIC at 10μg/mL	W	44.0000	0.4494*	
	Wilcoxon rank sum test with continuity correction	SAC vs NIC at 50μg/mL	W	37.0000	0.2319*	
	Wilcoxon rank sum test with continuity correction	SAC vs NIC at 100μg/mL	W	49.0000	0.5818*	
	Wilcoxon rank sum test with continuity correction	SAC vs NIC at 100μg/mL	W	73.0000	1.0000*	
**Open arm entries - adolescent pretreatment**						
SAC + SAL = 12, SAC + NIC = 16,	Shapiro-Wilk normality test		W	0.9280	0.0000	Fig S1I
NIC + SAL = 11, NIC + NIC = 14	Kruskal-Wallis rank sum test	All groups, 0-3 min	Kruskal-Wallis chi-squared	11.1952	0.0107	
	Kruskal-Wallis rank sum test	All groups, 3-6 min	Kruskal-Wallis chi-squared	11.7069	0.0085	
	Kruskal-Wallis rank sum test	All groups, 6-9 min	Kruskal-Wallis chi-squared	14.1702	0.0027	
	Wilcoxon rank sum test with continuity correction	NIC-SAL vs NIC-NIC, 0-3 min	W	53.5000	0.5292*	
	Wilcoxon rank sum test with continuity correction	SAC-SAL vs SAC-NIC, 0-3 min	W	38.0000	0.0279*	
	Wilcoxon rank sum test with continuity correction	SAC-SAL vs NIC-SAL, 0-3 min	W	48.0000	0.5292*	
	Wilcoxon rank sum test with continuity correction	SAC-NIC vs NIC-NIC, 0-3 min	W	144.5000	0.5292*	
	Wilcoxon rank sum test with continuity correction	NIC-SAL vs NIC-NIC, 3-6 min	W	35.0000	0.0707*	
	Wilcoxon rank sum test with continuity correction	SAC-SAL vs SAC-NIC, 3-6 min	W	45.5000	0.0707*	
	Wilcoxon rank sum test with continuity correction	SAC-SAL vs NIC-SAL, 3-6 min	W	77.0000	1.0000*	
	Wilcoxon rank sum test with continuity correction	SAC-NIC vs NIC-NIC, 3-6 min	W	122.0000	1.0000*	
	Wilcoxon rank sum test with continuity correction	NIC-SAL vs NIC-NIC, 6-9 min	W	49.5000	0.4067*	

* Holm correction for multiple comparisons

Supplementary Table 1

Detailed Statistics for Supplementary Figure 1

N	Test	Factor	Statistic	Statistic Value	p value	Corresponding Figure
	Wilcoxon rank sum test with continuity correction	SAC-SAL vs SAC-NIC, 6-9 min	W	26.5000	0.0043*	
	Wilcoxon rank sum test with continuity correction	SAC-SAL vs NIC-SAL, 6-9 min	W	83.5000	0.4663*	
	Wilcoxon rank sum test with continuity correction	SAC-NIC vs NIC-NIC, 6-9 min	W	140.5000	0.4663*	
Distance traveled EOM - adolescent pretreatment						
SAC + SAL = 12, SAC + NIC = 16,	Shapiro-Wilk normality test		W	0.9809	0.0265	Fig S1I
NIC + SAL = 11, NIC + NIC = 14	Kruskal-Wallis rank sum test	All groups, 0-3 min	Kruskal-Wallis chi-squared	14.4259	0.0024	
	Kruskal-Wallis rank sum test	All groups, 3-6 min	Kruskal-Wallis chi-squared	8.5321	0.0362	
	Kruskal-Wallis rank sum test	All groups, 6-9 min	Kruskal-Wallis chi-squared	6.7473	0.0804	
	Wilcoxon rank sum test with continuity correction	NIC-SAL vs NIC-NIC, 0-3 min	W	40.0000	0.1371*	
	Wilcoxon rank sum test with continuity correction	SAC-SAL vs SAC-NIC, 0-3 min	W	30.0000	0.0094*	
	Wilcoxon rank sum test with continuity correction	SAC-SAL vs NIC-SAL, 0-3 min	W	54.0000	0.8837*	
	Wilcoxon rank sum test with continuity correction	SAC-NIC vs NIC-NIC, 0-3 min	W	131.0000	0.8837*	
	Wilcoxon rank sum test with continuity correction	NIC-SAL vs NIC-NIC, 3-6 min	W	54.0000	0.6541*	
	Wilcoxon rank sum test with continuity correction	SAC-SAL vs SAC-NIC, 3-6 min	W	39.0000	0.0349*	
	Wilcoxon rank sum test with continuity correction	SAC-SAL vs NIC-SAL, 3-6 min	W	79.0000	0.8834*	
	Wilcoxon rank sum test with continuity correction	SAC-NIC vs NIC-NIC, 3-6 min	W	130.0000	0.8834*	
	Wilcoxon rank sum test with continuity correction	NIC-SAL vs NIC-NIC, 6-9 min	W	57.0000	0.8572*	
	Wilcoxon rank sum test with continuity correction	SAC-SAL vs SAC-NIC, 6-9 min	W	49.0000	0.1235*	

* Holm correction for multiple comparisons

Supplementary Table 1

Detailed Statistics for Supplementary Figure 1

N	Test	Factor	Statistic	Statistic Value	p value	Corresponding Figure
	Wilcoxon rank sum test with continuity correction	SAC-SAL vs NIC-SAL, 6-9 min	W	71.0000	0.8572*	
	Wilcoxon rank sum test with continuity correction	SAC-NIC vs NIC-NIC, 6-9 min	W	138.0000	0.8572*	
**Open arm entries - adult pretreatment**						
SAC + SAL = 15, SAC + NIC = 15,	Shapiro-Wilk normality test		W	0.8975	0.0000	Fig S1K
NIC + SAL = 15, NIC + NIC = 16	Kruskal-Wallis rank sum test	All groups, 0-3 min	Kruskal-Wallis chi-squared	25.7673	0.0000	
	Kruskal-Wallis rank sum test	All groups, 3-6 min	Kruskal-Wallis chi-squared	20.4812	0.0001	
	Kruskal-Wallis rank sum test	All groups, 6-9 min	Kruskal-Wallis chi-squared	22.0303	0.0001	
	Wilcoxon rank sum test with continuity correction	NIC-SAL vs NIC-NIC, 0-3 min	W	100.5000	0.0792*	
	Wilcoxon rank sum test with continuity correction	SAC-SAL vs SAC-NIC, 0-3 min	W	47.0000	0.0792*	
	Wilcoxon rank sum test with continuity correction	SAC-SAL vs NIC-SAL, 0-3 min	W	149.5000	0.3767*	
	Wilcoxon rank sum test with continuity correction	SAC-NIC vs NIC-NIC, 0-3 min	W	284.5000	0.0938*	
	Wilcoxon rank sum test with continuity correction	NIC-SAL vs NIC-NIC, 3-6 min	W	86.5000	0.0172*	
	Wilcoxon rank sum test with continuity correction	SAC-SAL vs SAC-NIC, 3-6 min	W	78.5000	0.0020*	
	Wilcoxon rank sum test with continuity correction	SAC-SAL vs NIC-SAL, 3-6 min	W	181.0000	1.0000*	
	Wilcoxon rank sum test with continuity correction	SAC-NIC vs NIC-NIC, 3-6 min	W	223.0000	1.0000*	
	Wilcoxon rank sum test with continuity correction	NIC-SAL vs NIC-NIC, 6-9 min	W	69.0000	0.0043*	
	Wilcoxon rank sum test with continuity correction	SAC-SAL vs SAC-NIC, 6-9 min	W	86.0000	0.0043*	
	Wilcoxon rank sum test with continuity correction	SAC-SAL vs NIC-SAL, 6-9 min	W	188.5000	1.0000*	

* Holm correction for multiple comparisons

Supplementary Table 1

Detailed Statistics for Supplementary Figure 1

N	Test	Factor	Statistic	Statistic Value	p value	Corresponding Figure
	Wilcoxon rank sum test with continuity correction	SAC-NIC vs NIC-NIC, 6-9 min	W	224.0000	1.0000*	
**Distance traveled EOM - adult pretreatment**						
SAC + SAL = 15, SAC + NIC = 15,	Shapiro-Wilk normality test		W	0.9697	0.0001	Fig S1K
NIC + SAL = 15, NIC + NIC = 16	Kruskal-Wallis rank sum test	All groups, 0-3 min	Kruskal-Wallis chi-squared	26.7687	0.0000	
	Kruskal-Wallis rank sum test	All groups, 3-6 min	Kruskal-Wallis chi-squared	21.1452	0.0001	
	Kruskal-Wallis rank sum test	All groups, 6-9 min	Kruskal-Wallis chi-squared	9.8571	0.0198	
	Wilcoxon rank sum test with continuity correction	NIC-SAL vs NIC-NIC, 0-3 min	W	87.0000	0.0205*	
	Wilcoxon rank sum test with continuity correction	SAC-SAL vs SAC-NIC, 0-3 min	W	42.0000	0.0000*	
	Wilcoxon rank sum test with continuity correction	SAC-SAL vs NIC-SAL, 0-3 min	W	173.0000	0.8493*	
	Wilcoxon rank sum test with continuity correction	SAC-NIC vs NIC-NIC, 0-3 min	W	260.0000	0.3934*	
	Wilcoxon rank sum test with continuity correction	NIC-SAL vs NIC-NIC, 3-6 min	W	86.0000	0.0188*	
	Wilcoxon rank sum test with continuity correction	SAC-SAL vs SAC-NIC, 3-6 min	W	74.0000	0.0016*	
	Wilcoxon rank sum test with continuity correction	SAC-SAL vs NIC-SAL, 3-6 min	W	189.0000	1.0000*	
	Wilcoxon rank sum test with continuity correction	SAC-NIC vs NIC-NIC, 3-6 min	W	223.0000	1.0000*	
	Wilcoxon rank sum test with continuity correction	NIC-SAL vs NIC-NIC, 6-9 min	W	118.0000	0.2165*	
	Wilcoxon rank sum test with continuity correction	SAC-SAL vs SAC-NIC, 6-9 min	W	114.0000	0.0510*	
	Wilcoxon rank sum test with continuity correction	SAC-SAL vs NIC-SAL, 6-9 min	W	194.0000	1.0000*	
	Wilcoxon rank sum test with continuity correction	SAC-NIC vs NIC-NIC, 6-9 min	W	216.0000	1.0000*	

* Holm correction for multiple comparisons

Supplementary Figure 2. *Supplementary data for Figure 2*. (**A**) Experimental strategy. (**B**) Volcano plots showing differential cFos expression across brain regions following an injection of saline for adolescent-pretreated (*Left*) and adult-pretreated mice (*Right*). (**C-D**) Correlation matrices for relationships between cFos expression in brain regions following an injection of saline in adolescent-pretreated (**C**) and adult-pretreated mice (**D**). (**E**) Correlation matrices for relationships between cFos expression in brain regions following an injection of nicotine in adult-pretreated mice. (**F**) Voxel-by-voxel analysis reveals no change in reactivity to nicotine within the VTA and DA terminal regions in adult-pretreated mice. (**G**) Full networks derived from correlation matrices in Fig 2C and organized into communities with Louvain community analysis. Source data are provided as a Source Data file.

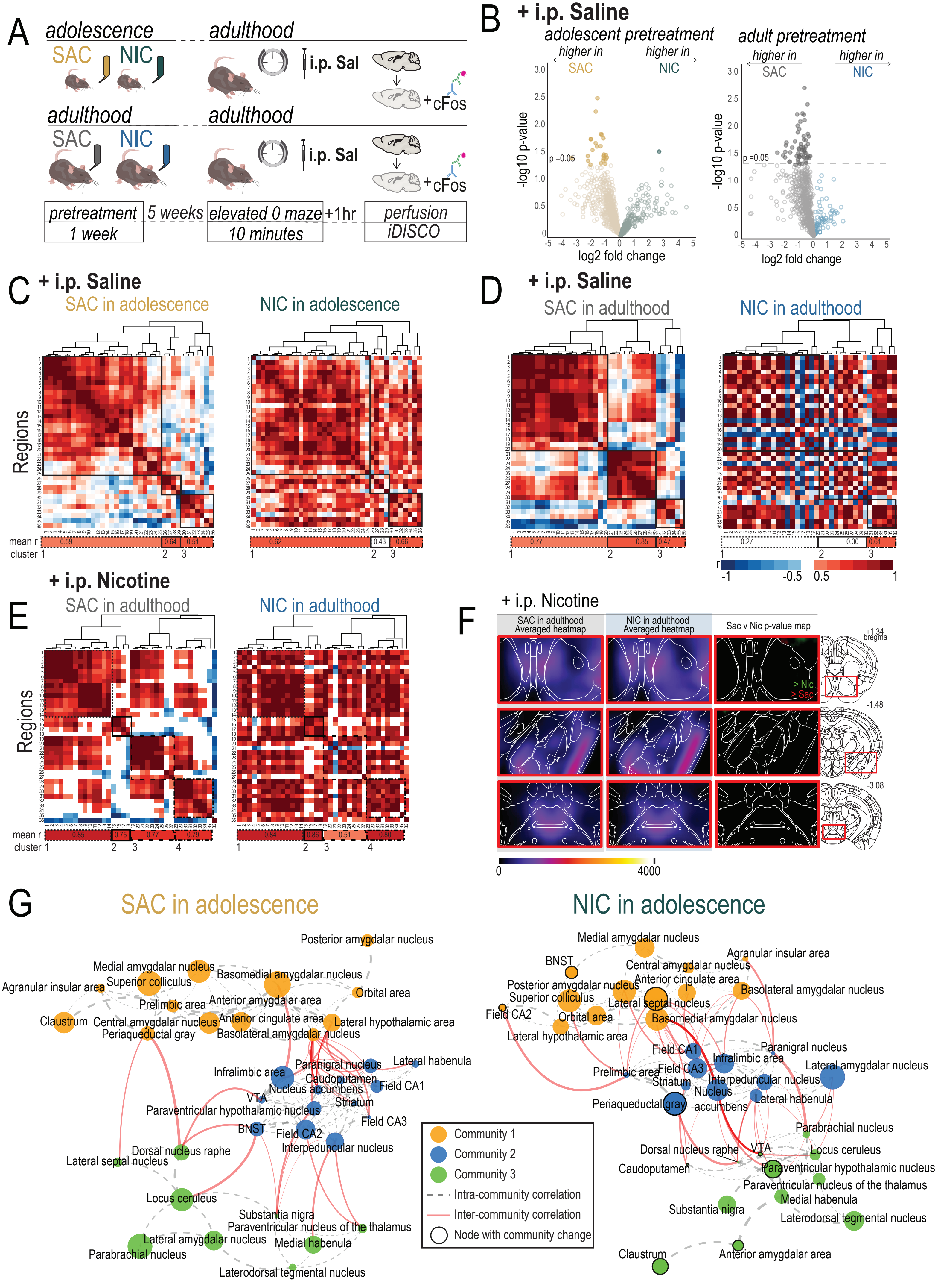

Supplementary Figure 2

Supplementary Figure 3. *Supplementary data for Figure 3*. (**A**) Experimental design for DA terminal density analysis. (**B**) Tyrosine Hydroxylase (TH)+ labeling in DA terminal regions. (Core = NAc Core, MShell = NAc Medial Shell, LShell = NAc Lateral Shell, CeA = Central Amygdala, BLA = Basolateral Amygdala, CPu = Caudate-Putamen). (**C**) Confocal image processing for bouton segmentation. (**D**) Bouton density counts across the NAc (*Left*) and the AMG (*Right*). (**E**) *Left* : Nicotine current traces were analyzed and neurons were found to show two types of responses to nicotine: monophasic (one component) and bi-phasic (with a fast and slow component). More neurons from adult mice pretreated with NIC in adolescence showed a biphasic response than those treated with SAC. *Center*: Nicotine currents in neurons from mice treated with NIC in adolescence showed a lower τ indicative of a faster current decay. *Right*: Less charge is transferred in response to a nicotine puff in neurons from mice treated with NIC in adolescence. (**F**) Average change in NMDAR currents following application of Ifenprodil (5 mM) to VTA DA neurons (SAC N = 4, n = 7; NIC N = 4, n = 7). (**G**) Paired pulse ratio measurements for VTA DA neurons of adolescent-pretreated (*Top*) and adult-pretreated mice (*Bottom*). (**H**) sEPSCs onto DA neurons measured before and after nicotine puff (*Left*) sEPSC frequency was reduced at baseline and after nicotine puff in adult mice pretreated with NIC in adolescence in comparison to mice pretreated with SAC (*Top row*), however NIC pretreated mice showed a greater augmentation (% of baseline) in response to the nicotine puff (*Right*). Mice pretreated with NIC in adulthood showed no differences from mice pretreated with SAC in adulthood (*Bottom row*). All bar graphs are presented as mean values ± SEM. *p < 0.05, **p < 0.01, ***p < 0.01. Detailed Statistics are available in Supplementary Table 5. Source data are provided as a Source Data file.

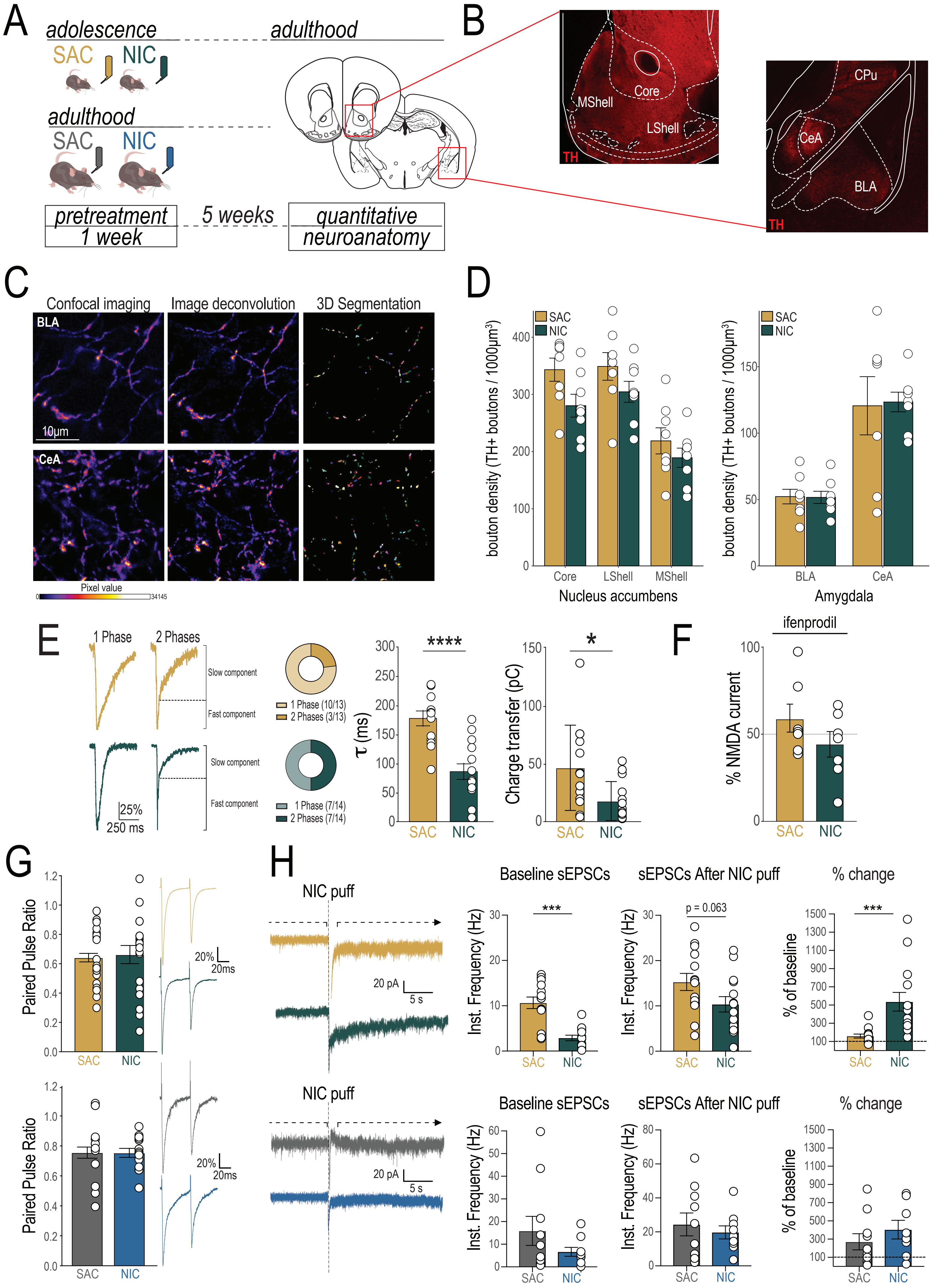

Supplementary Figure 3

Supplementary Table 2

Detailed Statistics for Supplementary Figure 3

N	Test	Factor	Statistic	Statistic Value	p value	Corresponding Figure
**NAc boutons**						
SAC = 8, NIC = 8	Shapiro-Wilk normality test		W	0.9719	0.3007	Fig S3D
	Two-way mixed ANOVA	Interaction	F	1.1643	0.3268	
		Treatment (between subj)	F	3.0443	0.1029	
		Region (within subjects)	F	74.5824	0.0000	
**Amg boutons**						
SAC = 7, NIC = 8	Shapiro-Wilk normality test		W	0.8895	0.0040	Fig S3D
	Wilcoxon rank sum test with continuity correction	SAC vs NIC in the BLA	W	34.0000	1.0000[1]	
	Wilcoxon rank sum test with continuity correction	SAC vs NIC in the CeA	W	31.0000	1.0000[1]	
	Wilcoxon rank sum test with continuity correction	BLA vs CeA in SAC	W	11.0000	0.1686[1]	
	Wilcoxon rank sum test with continuity correction	BLA vs CeA in NIC	W	0.0000	0.0038[1]	
**Weighted Tau, nicotine current**						
NIC = 2 mice, 14 neurons; SAC = 2 mice, 13 neurons	Shapiro-Wilk normality test		W	0.9567	0.3102	Fig S3E
	Welch Two Sample t-test	SAC vs NIC	t	-6.7097	0.0000	
**Charge Transferred - nicotine current**						
NIC = 2 mice, 14 neurons; SAC = 2 mice, 13 neurons	Shapiro-Wilk normality test		W	0.8268	0.0004	Fig S3E
	Wilcoxon rank sum test with continuity correction	SAC vs NIC	W	141.0000	0.0163	
**% NMDA current - ifenprodil**						
NIC = 4 mice, 7 neurons; SAC = 4 mice, 7 neurons	Shapiro-Wilk normality test		W	0.9679	0.8470	Fig S3F
	Welch Two Sample t-test	SAC vs NIC	t	-1.3859	0.1912	

[1] Holm correction for multiple comparisons

Supplementary Table 2

Detailed Statistics for Supplementary Figure 3

N	Test	Factor	Statistic	Statistic Value	p value	Corresponding Figure
**PPR - adolescent pretreatment**						
NIC = 5 mice, 20 neurons; SAC = 5 mice, 19 neurons	Shapiro-Wilk normality test		W	0.9746	0.5122	Fig S3G
	Welch Two Sample t-test	SAC vs NIC	t	0.3017	0.7653	
**PPR - adult pretreatment**						
NIC = 4 mice, 12 neurons; SAC = 4 mice, 14 neurons	Shapiro-Wilk normality test		W	0.9705	0.6154	Fig S3G
	Welch Two Sample t-test	SAC vs NIC	t	-0.0377	0.9702	
**Baseline sEPSCs - adolescent pretreatment**						
NIC = 2 mice, 14 neurons; SAC = 2 mice, 14 neurons	Shapiro-Wilk normality test		W	0.9020	0.0127	Fig S3H
	Wilcoxon rank sum test with continuity correction	SAC vs NIC	W	18.0000	0.0003	
**After sEPSCs - adolescent pretreatment**						
NIC = 2 mice, 14 neurons; SAC = 2 mice, 14 neurons	Shapiro-Wilk normality test		W	0.9656	0.4681	Fig S3H
	Welch Two Sample t-test	SAC vs NIC	t	-1.9394	0.0635	
**Percent sEPSCs - adolescent pretreatment**						
NIC = 2 mice, 14 neurons; SAC = 2 mice, 14 neurons	Shapiro-Wilk normality test		W	0.7356	0.0000	Fig S3H
	Wilcoxon rank sum test with continuity correction	SAC vs NIC	W	183.0000	0.0001	
**Baseline sEPSCs - adult pretreatment**						
NIC = 5 mice, 8 neurons; SAC = 5 mice, 10 neurons	Shapiro-Wilk normality test		W	0.6963	0.0001	Fig S3H
	Wilcoxon rank sum test with continuity correction	SAC vs NIC	W	39.0000	0.6534	
**After sEPSCs - adult pretreatment**						
NIC = 5 mice, 8 neurons; SAC = 5 mice, 10 neurons	Shapiro-Wilk normality test		W	0.9171	0.1001	Fig S3H

[1] Holm correction for multiple comparisons

Supplementary Table 2

Detailed Statistics for Supplementary Figure 3

N	Test	Factor	Statistic	Statistic Value	p value	Corresponding Figure
	Welch Two Sample t-test	SAC vs NIC	t	-0.6067	0.5536	
**Percent sEPSCs - adult pretreatment**						
NIC = 5 mice, 8 neurons; SAC = 5 mice, 10 neurons	Shapiro-Wilk normality test		W	0.8870	0.0343	Fig S3H
	Wilcoxon rank sum test with continuity correction	SAC vs NIC	W	52.0000	0.3069	

[1] Holm correction for multiple comparisons
